# Supplementary material for: A Global Screen for Assembly State Changes of the Mitotic Proteome by SEC-SWATH-MS
Source: Cell Syst. 2020 Feb 26;10(2):133–155.e6. doi: 10.1016/j.cels.2020.01.001 (PMC7042714; doi:10.1016/j.cels.2020.01.001)

P57105 | SYJ2B_HUMAN | SYNJ2BP OMP25
Monomer MW [kDa]: 15.928 Monomer expected elution fraction: 56

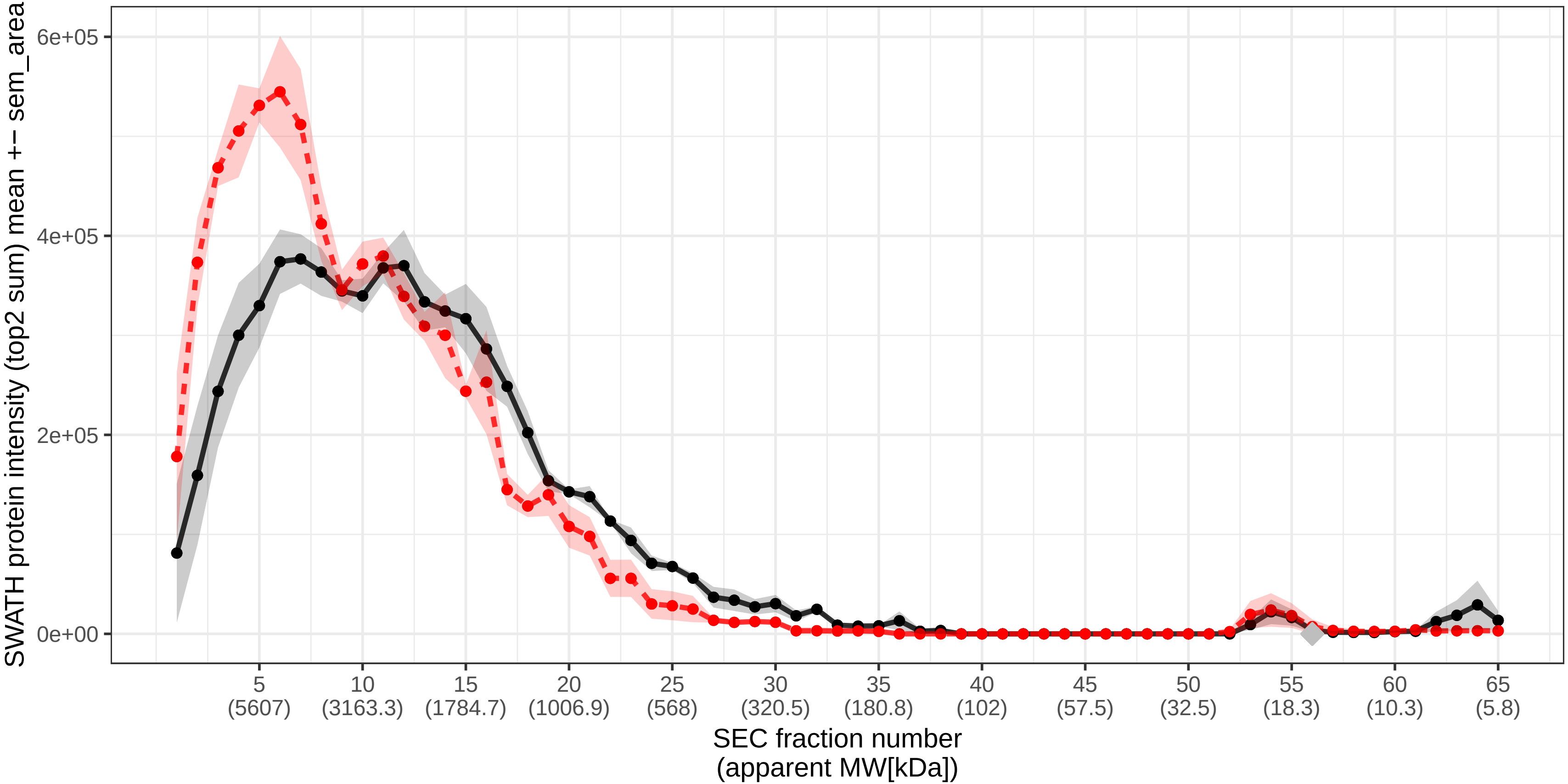

Supplement: Data S1. SEC-SWATH-MS Protein Chromatograms, Related to Figure 1 [file mmc6.zip › SECchrom_P57105_SYJ2B_HUMAN_SYNJ2BP_OMP25.pdf]
